# Reduced Expression of *METTL3* Promotes Metastasis of Triple-Negative Breast Cancer by m6A Methylation-Mediated *COL3A1* Up-Regulation

**DOI:** 10.3389/fonc.2020.01126

**Published:** 2020-07-14

**Authors:** Yu Shi, Chunlei Zheng, Yue Jin, Bowen Bao, Duo Wang, Kezuo Hou, Jing Feng, Shiying Tang, Xiujuan Qu, Yunpeng Liu, Xiaofang Che, Yuee Teng

**Affiliations:** ^1^Department of Medical Oncology, The First Hospital of China Medical University, Shenyang, China; ^2^Key Laboratory of Anticancer Drugs and Biotherapy of Liaoning Province, The First Hospital of China Medical University, Shenyang, China; ^3^Liaoning Province Clinical Research Center for Cancer, China Medical University, Shenyang, China

**Keywords:** *METTL3*, m6A, triple-negative breast cancer, metastasis, *COL3A1*

## Abstract

The abnormal m6A modification caused by m6A modulators is a common feature of various tumors; however, little is known about which m6A modulator plays the most important role in triple-negative breast cancer (TNBC). In this study, when analyzing the influence of m6A modulators (*METTL3, METTL14, WTAP, FTO*, and *ALKBH5*) on the prognosis of breast cancer, especially in TNBC using several on-line databases, methyltransferase-like 3 (*METTL3*) was found to have low expression in breast cancer, and was closely associated with short-distance-metastasis-free survival in TNBC. Further investigation showed that knockdown of *METTL3* could enhance the ability of migration, invasion, and adhesion by decreasing m6A level in TNBC cell lines. Collagen type III alpha 1 chain (*COL3A1*) was identified and verified as a target gene of *METTL3*. *METTL3* could down-regulate the expression of *COL3A1* by increasing its m6A methylation, ultimately inhibiting the metastasis of TNBC cells. Finally, with immunohistochemistry staining in breast cancer tissues, it was proved that *METTL3* expression was negatively correlated with *COL3A1* in TNBC, but not in non-TNBC. This study demonstrated the potential mechanism of m6A modification in metastasis and provided potential targets for treatment in TNBC.

## Introduction

Breast cancer, the most common cancer in women, poses a serious threat to the health of women (1). Despite the improvement of treatment strategies, the prognosis of breast cancer, especially triple-negative breast cancer (TNBC), remains poor due to metastasis. In recent years, epigenetic regulation, such as DNA methylation, histone acetylation, and non-coding RNAs, has been reported to play a critical role in the development of breast cancer including TNBC. Especially, as a new emerging epigenetic modification, RNA methylation has attracted much attention due to its non-negligible function in cancer development; however, current studies of RNA methylation-related cancer development are just the tip of the iceberg in this cognate area. It is necessary to clarify the mechanisms underlying RNA methylation-involved metastasis in TNBC.

As the most prevalent RNA methylation modification, N6-methyladenosine (m6A) infers that the nitrogen-6 position of adenosine base in RNA is methylated by the regulation of m6A methyltransferases and m6A demethylases. Methyltransferase, including methyltransferase-like 3 (*METTL3*), methyltransferase-like 14 (*METTL14*), and Wilms tumor 1-associated protein (*WTAP*) can form into complexes and mediate the cellular deposition of m6A on mammalian mRNAs, whereas demethylases including *FTO* and its homolog AlkB family member 5 (*ALKBH5*) can selectively reverse m6A to adenosine (2–5). RNA m6A methylation is known to be involved in various biological processes, such as stem cell differentiation and pluripotency, circadian periods, embryogenesis, and DNA damage response (2, 3). Cumulative studies have proved that the change of RNA m6A modification caused by the aberrant expression of m6A modulators can also influence the development of cancers. The methyltransferase *METTL14* can inhibit tumor metastasis in HCC by positively regulating the m6A level of *DGCR8* and promoting the binding of *DGCR8* to pri-miRNAs (6); similarly, the demethylase *FTO* can promote cell proliferation via down-regulating the m6A level of *USP7* in advanced non-small cell lung cancer, indicating the repression role of m6A in cancer development (7), however, other studies obtained contradictory results wherein *METTL3* could promote the proliferation of prostate cancer cell via enhancing the m6A level of *GLI15* (8); similarly, *ALKBH5* was found to be able to inhibit pancreatic cancer metastasis by down-regulating *KCNK15-AS1*, suggesting that m6A modification of RNA plays an oncogenic role in cancer (9). Therefore, it seems that an m6A modulator might play both promotional, and inhibitory roles in different types of cancers by regulating different specific target genes. To date, the role of RNA m6A methylation in the development of breast cancer remains unclear. The only studies of breast cancer have shown that *METTL3*-mediated enhancement of m6A level could promote the proliferation of breast cancer cells (10), while the high level of m6A caused by *FTO* knockdown could inhibit the proliferation and metastasis of breast cancer (11). Therefore, it seems that, although the changes in m6A level are consistent, the effects of different modulators on breast cancer differ because the specific target genes are different. The underlying role and epigenetic regulation of m6A modulators in breast cancer, especially in TNBC, still needs to be investigated.

In this study, we analyzed the prognostic role of m6A modulators in TNBC using several online databases and found that the low expression of *METTL3* was associated with the poor prognosis of TNBC. Further molecular mechanism investigation indicated that silence of *METTL3* could up-regulate the expression of Collagen type III alpha 1 chain (*COL3A1*) by increasing m6A-levels, ultimately promoting metastasis of TNBC cells. This study revealed the important role of m6A modification mediated by *METTL3* in TNBC and suggests that *METTL3* might act as a novel therapeutic target in TNBC metastasis.

## Materials and Methods

### Data Sources: On-Line Databases

KM plotter (http://kmplot.com/analysis/) is a website used for on-line analysis, which is capable of assessing 54k genes on the survival of 21 cancer types, including breast cancer. The association between the distance-metastasis-free survival (DMFS) and the expression of m6A modulators (*METTL3, METTL14, WTAP, FTO*, and *ALKBH5*), *COL3A1* was analyzed using KM plotter, respectively. GEPIA (http://gepia.cancer-pku.cn) is an on-line database including the RNA sequencing expression data of 9,736 tumors and 8,587 normal tissue samples from the Cancer Genome Atlas (TCGA) and the Genotype Tissue Expression (GTEx). The transcriptional levels of five m6A modulators above in breast cancer tissues and normal breast tissues were obtained from GEPIA. The mRNA expression data of 91 patients with TNBC were downloaded from TCGA (https://www.cancer.gov/) for analysis of the correlation between the m6A modulators and target genes.

### Cell Culture

The human breast cancer cell lines, MDA-MB-231, and MDA-MB-468, were acquired from the Cell Bank of the Chinese Academy of Sciences (Shanghai, China). All the cells were incubated in L15 culture medium (Gibco, NY, USA) supplemented with 10% FBS at 37°C under 5% CO_2_ and saturated humidity.

### Antibodies

The primary antibodies for western blot, anti-*METTL3* (#96391) were sourced from Cell Signaling Technology (MA, USA), anti-*COL3A1* (sc-514601) was sourced from Santa Cruz (CA, USA), anti-α-tubulin (ab7291) was sourced from Abcam (CA, USA). HRP-conjugated goat anti-mouse/rabbit secondary antibodies (ZDR-5306/5307) were sourced from ZSBIO (Beijing, China). The antibodies for immunohistochemistry, anti-*METTL3* (ab195352) were sourced from Abcam (CA, USA), and anti-*COL3A1* (sc-166316) was sourced from Santa Cruz (CA, USA).

### Trans-well Migration and Invasion Assays

For the migration assay, ~2 × 10^4^ cells were suspended in 200 μl serum-free L15 medium and added into the upper chamber of a trans-well plate (Corning, USA) with an 8-μm pore size polycarbonate filter, and 500 μl L15 medium with 10% FBS were dispensed into the lower chambers, and incubated for 24 h. Then the upper chambers were fixed in 75% ethanol, and the cells on the upper surface of the filter were removed manually with a cotton swab. Then the migrating cells were stained with Wright–Giemsa stain.

The invasion assay was similar to the migration assay except that 3% matrigel was dispensed into the upper chamber before seeding 3 × 10^4^ cells into the culture system. Migrating and invading cells were observed under an optical microscope. The cells from three fields were counted with Image J (https://imagej.nih.gov/ij/download.html).

### Cell Adhesion Assay

The cells were seeded at 3 × 10^4^ per well into 96-well-plates pre-coated with 10% matrigel overnight. After 30 min-incubation at 37 °C, the non-adherent cells were removed by PBS washing. Then the cells were fixed with 75% ethanol for 10 min and stained with Wright–Giemsa stain. Images were acquired by microscope and the quantities of cells were counted with Image J.

### RNA Isolation and Quantitative Real-Time PCR Assay

RNA was isolated with TRIzol reagent (Invitrogen, CA, USA) and identified using a NanoDrop spectrophotometer (Thermo Scientific, Rockford, IL, USA). The Reverse Transcription Kit (Promega, WI, USA) was used for mRNA reverse transcription. Quantitative real-time PCR (qRT-PCR) assay was then performed using the SYBR Green kit (Promega, WI, USA) on the ABI7500 instrument (Thermofisher, IL, USA). All the reactions were conducted for triplicates. 18S RNA was used as the internal control. Primers of *METTL3, COL3A1, MYH11*, and 18S were used as follows:

*METTL3* forward: 5′-TTGTCTCCAACCTTCCGTAGT-3′
*METTL3* reverse: 5′-CCAGATCAGAGAGGTGGTGTAG-3′
*COL3A1* forward: 5′-CCCACTATTATTTTGGCACAACAG-3′
*COL3A1* reverse: 5′-AACGGATCCTGAGTCACAGACA-3′
*MYH11* forward: 5′-AGTTCGAAAGGGATCTCCA-3′
*MYH11* reverse: 5′-CATACTCGTGAAGCTGTCTC-3′
18S forward: 5′CCCGGGGAGGTAGTGACGAAAAAT-3′
18S reverse: 5′-CGCCCGCCCGCTCCCAAGAT-3′.

### Methylated RNA Immunoprecipitation-qRT-PCR

The methylated RNA immunoprecipitation-qRT-PCR (MeRIP-qRT-PCR) assay was conducted according to the standard protocol of Magna MeRIP m6A Kit (Millipore, MA, USA, 17-10499) with a slight modification. The total RNA (300 μg) was isolated with TRIzol reagent and fragmented. Except for 3 μg of the total RNA as input, the remaining RNA was used for m6A-immunoprecipitation with m6A antibody. The Protein A/G Magnetic Beads were prepared by 30 min-incubation with m6A-specific antibody in immunoprecipitation buffer at room temperature, then incubated with the MeRIP reaction mixture and RNA for 2h at 4°C. Finally, the MeRIPed-RNA was cleaned up and concentrated with RNeasy MinElute Clean-up Kit (Qiagen, Hilden, Germany). The enriched RNA level of *COL3A1* was analyzed by qRT-PCR. The primers were as same as the primers used in real-time PCR assay.

### Transfection

For the knockdown of *METTL3* and *COL3A1*, the cells were seeded at 10^5^ cells/well in a 6-well-plate and the siRNAs targeted to *METTL3* and *COL3A1* (GENEWIZ, Beijing, China) at a final concentration of 50 nM were transfected using JetPRIME Transfection Reagent (Polyplus, Illkirch, France) according to the manufacturer's instructions. Negative control (NC) siRNA was used as a control. For the overexpression of *METLL3*, the cells were seeded at 10^5^ per 6-well-plate and transfected with plasmids pcDNA3.1-FLAG and pcDNA3.1-*METTL3* (Obio, Shanghai, China) at a final concentration of 1 μg/ml using JetPRIME Transfection Reagent. The siRNA sequences of NC, *METTL3*, and *COL3A1* were used as follows:

si-*METTL3*-1126 (KD1): 5′-CCUGCAAGUAUGUUCACUATT-3′
si-*METTL3*-1400 (KD2): 5′-GCUCAACAUACCCGUACUATT-3′
si-*METTL3*-1604 (KD3): 5′-GGUUGGUGUCAAAGGAAAUTT-3′
si-*COL3A1*-1 (KD1): 5′-GGAUGCAAAUUGGAUGCUAtt-3′
si-*COL3A1*-2 (KD2): 5′-CCCUCCUAAUGGUCAAGGAtt-3′
si-NC: 5′-UUCUCCGAACGUGUCACGUtt-3′.

### Western Blot

The harvested cells were lysed in a lysis buffer (50 mM Tris, pH 7.4, 150 mM NaCl, 10 mM EDTA, 50 mM NaF, 1 mM NaVanadate, 1% Triton X-100, 1 mM PMSF, and 0.5% aprotin) and the protein concentration was quantified according to the Coomassie blue G250 staining technique. Equivalent protein was electrophoresed on 8–10% SDS-PAGE gels and transferred to polyvinylidene difluoride (PVDF) membranes (Perkin-Elmer, Waltham, MA, USA). Then the membranes were blocked with 5% skimmed milk in TBST for 1 h and incubated with the primary antibodies overnight (more than 6 h). The concentration of anti-*METTL3* (#96391) was 1:1,000, the concentration of anti-*COL3A1* (sc-514601) was 1:200, the concentration of anti-α-tubulin (ab7291) was 1:1,000. After being immunoblotted with HRP-conjugated goat anti-mouse/rabbit antibody (1:2,000) for 40 min, the signal strength of revealed protein bands could be detected with enhanced chemiluminescence reagent (SuperSignal Western Pico Chemiluminescent Substrate; Pierce, Rockford, IL, USA) and visualized with the Electrophoresis Gel Imaging Analysis System (DNR Bio-Imaging Systems, Jerusalem, Israel). The blots were scanned and the abundance assessed quantitatively using ImageJ.

### ELISA

Cells at a density of 1 × 10^5^/well in a 6-well-plate were incubated for 72 h, and Collagen α1(III) levels in cell culture medium and cell lysate were measured, respectively, using a commercially available Collagen α1(III) ELISA kit at 450 nm by a microplate spectrophotometer (CSB-E13446h, Cusabio, Wuhan, China). The experiment process was carried out according to the instructions of the kit. The samples were added into wells and incubated 2 h in 37°C. After removing the liquid of each well, Biotin-antibody was added into each well. After incubated 1 h in 37°C, HRP-avidin and TMB Substrate were used for color rendering. The standard curve was constructed by the mean absorbance of each standard and the concentration. The concentration of each sample was determined according to standard curve. To acquire the total amount of Collagen α1(III) in each sample, the concentration was multiplied by the total volume. Then the amount of Collagen α1(III) in each sample was normalized to the amount of Collagen α1(III) in 10^7^ cells. All samples and standards were detected in duplicate.

### Immunohistochemistry

The tissue microarray (TMA) sections (HBreD140Su06) and the relevant clinical data were obtained from Shanghai Outdo Biotechnology Company. This study was approved by the Ethics Committee of Shanghai Outdo Biotechnology Company (YB M-05-02), and all patients have given their informed consent. The TMA sections were deparaffinized and rehydrated with an ethanol gradient. Then antigen retrieval was performed with citrate buffer (MXB, Fuzhou, China, MVS-0066) and the TMA sections were blocked with endogenous peroxidases in UltraSensitive™ SP (Mouse/Rabbit) IHC Kit (MXB, Fuzhou, China, KIT-9730-A&B). The concentration of anti-*METTL3* (ab195352) was 1:500, the concentration of anti-*COL3A1* (sc-166316) was 1:50. After overnight incubation with primary antibody, the TMA sections were incubated with biotinylated secondary antibody for 10 min in UltraSensitive™ SP (Mouse/Rabbit) IHC Kit (MXB, Fuzhou, China, KIT-9730-C&D) and developed with DAB Kit (MXB, China, DAB-0031). Finally, the TMA sections were counterstained with hematoxylin (Solarbio, Beijing, China), and dehydrated with an ethanol gradient and mounted with neutral balsam (Solarbio, Beijing, China). *METTL3* expression was evaluated by two independent reviewers by calculating the average positively stained tumor cells at 400×magnification. The positive signal of *COL3A1* was quantified as integrate optical density (IOD) value using ImageJ software.

### Statistical Analysis

Statistical analyses were carried out using SPSS (version 16.0) and R (V.3.2.5). Limma package analysis was conducted to explore the correlations between *METTL3* and other genes. One-way ANOVA and Student's *t*-test were used to determine statistical significance. Statistical significance was identified as *P*-values of <0.05.

## Results

### Low Expression of *METTL3* Was Associated With Poor Prognosis in TNBC

**Figure 1:**
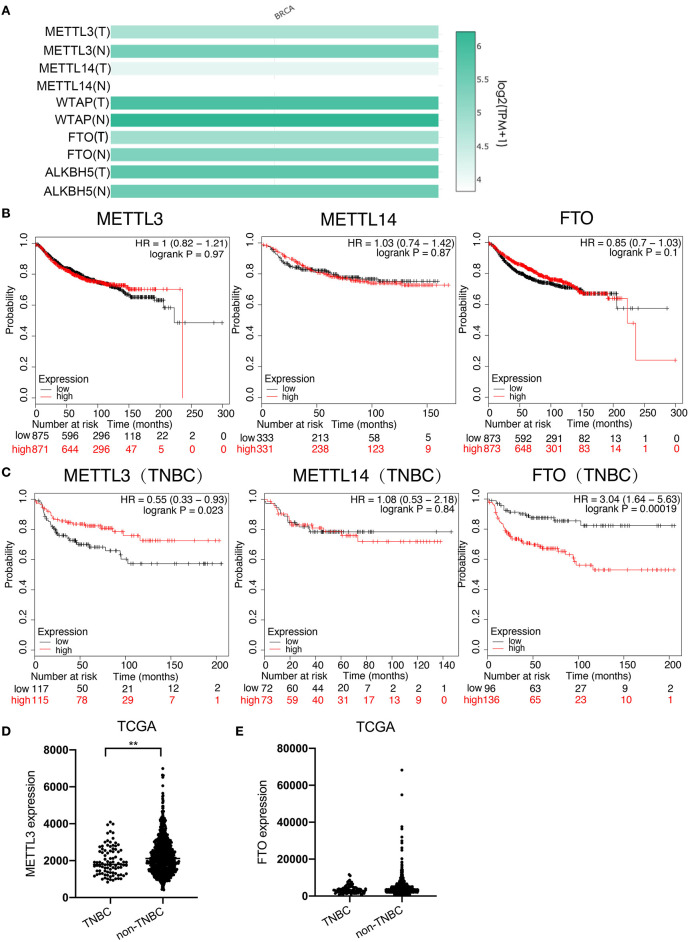
Low expression of *METTL3* was associated with poor prognosis in TNBC. **(A)** Expression analysis of *METTL3, METTL14, WTAP, FTO*, and *ALKBH5* in BC tissue (1085) and normal tissue (291) using TCGA and GTEx online database. **(B)** Kaplan–Meier analysis for the DMFS of *METTL3, METTL14*, and *FTO* in overall BC patients using KM-plotter online database. **(C)** Kaplan–Meier analysis for the DMFS of *METTL3, METTL14*, and *FTO* in TNBC patients using KM-plotter online database. **(D)** *METTL3* expression analysis in TNBC patients (*n* = 91) and non-TNBC patients (*n* = 1,005) using TCGA. **(E)** *FTO* expression analysis in TNBC patients (*n* = 1,005) and non-TNBC patients (*n* = 584) using TCGA. **P < 0.01.

To investigate which m6A modulator plays an important role in breast cancer, especially in TNBC, the mRNA expressions of m6A methyltransferases (*METTL3, METTL14*, and *WTAP*) and demethylases (*FTO* and *ALKBH5*) were first compared between breast cancer tissues and normal breast tissues using the RNA sequencing expression data in GEPIA (http://gepia.cancer-pku.cn) on-line database. The result showed that the expression of *METTL3* (*T* = 4.8, *N* = 5.4) and *FTO* (*T* = 4.9, *N* = 5.3) was significantly lower, whereas that of *METTL14* (*T* = 4.2, *N* = 3.8) was higher in breast cancer tissues than that in normal tissues, but the difference in the expression of *WTAP* (*T* = 5.9, *N* = 6.2) and *ALKBH5* (*T* = 5.7, *N* = 5.5) was not very significant (Figure 1A). Next, the effects of *METTL3, METTL14*, and *FTO* on distant metastasis free survival (DMFS) of total breast cancer patients and TNBC patients were analyzed using KM-plotter on-line database, respectively. For overall patients, the analysis result indicated that no significant difference of DMFS was obtained between the patients with all three modulators in high-expression groups and low-expression groups (Figure 1B); however, for the TNBC patients, although no significant difference was found between the DMFS of the *METTL14* high-expression group and low-expression group, the DMFS of *METTL3* high expression group was shown to be longer than that in the *METTL3* low-expression group, whereas the DMFS of the *FTO* high-expression group was shorter than that of the *FTO* low-expression group, indicating that *METTL3* is a protective factor, but *FTO* is a risk factor for DMFS of TNBC (Figure 1C). As the result, the low expression of *FTO* in TNBC tissues was contradictory to its role as a risk factor (Figures 1A,E) and only *METTL3* was shown to play an important inhibitory role in the metastasis of TNBC (Figures 1A,D), indicating that *METTL3* might contribute to the metastasis of TNBC. Therefore, the role of *METTL3* in TNBC metastasis was focused on in the following investigation.

### *METTL3* Suppressed Metastasis of TNBC Cells by Enhancing m6A Modification

**Figure 2:**
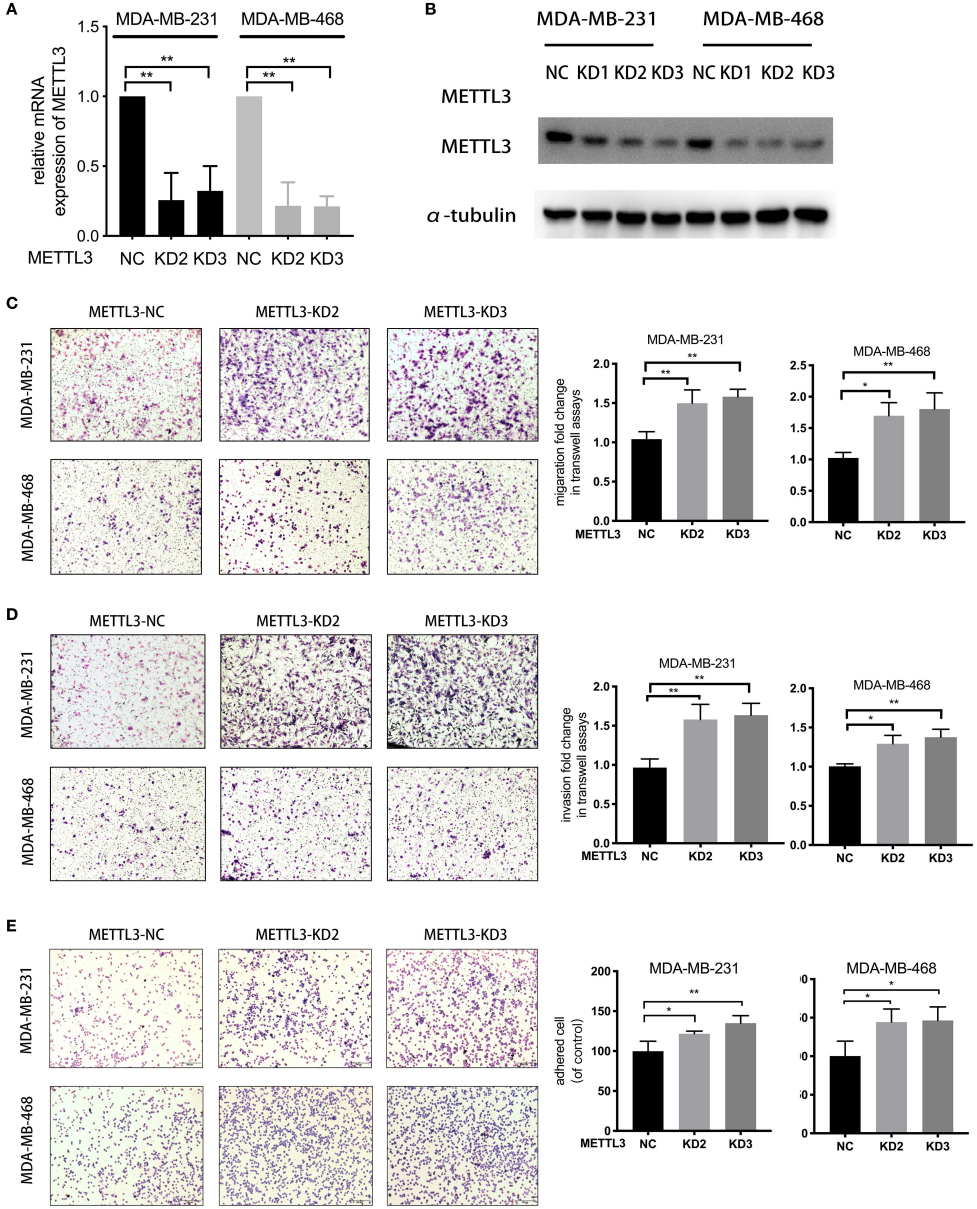
*METTL3* suppressed metastatic ability in TNBC cells. **(A)** qRT-PCR was used to detect *METTL3* expression in MDA-MB-231 and MDA-MB-468 cells transfected with si-NC or si-*METTL3*. 18S was used as an internal control. **(B)** Western blot was used to detect *METTL3* expression in MDA-MB-231 and MDA-MB-468 cells transfected with si-NC or si-*METTL3*. α-tubulin was used as a loading control. **(C,D)** Transwell assay was used to detect the migration and invasion ability in MDA-MB-231 and MDA-MB-468 cells with *METTL3* transient knockdown (left panels). Relative fold change was shown as the proportion of the number of control cells transfected with si-NC (right panels). Original magnification, 100×. **(E)** Adhesion assay was used to detect the adhesion ability of MDA-MB-231 and MDA-MB-468 cells with *METTL3* transient knockdown (left panels). Relative fold change was shown as the proportion of the number of control cells transfected with si-NC (right panels). Original magnification, 100×. *P < 0.05, **P < 0.01. Error bars represent the mean ± SD of three independent experiments.

**Figure 3:**
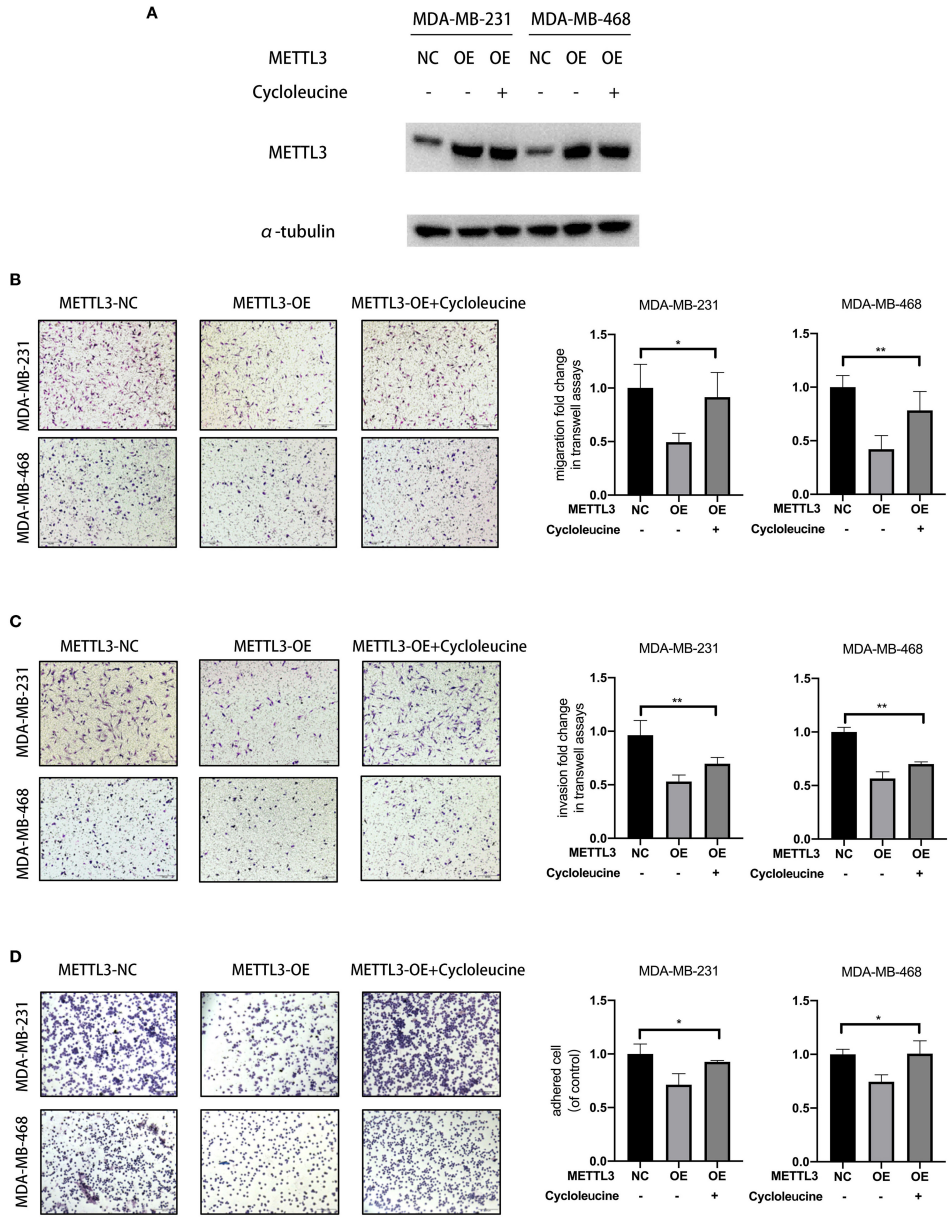
*METTL3* overexpression-suppressed migration, invasion, and adhesion were significantly rescued by cycloleucine. **(A)** Western blot was used to detect *METTL3* expression in MDA-MB-231 and MDA-MB-468 cells in rescued assay. α-tubulin was used as a loading control. **(B,C)** Transwell assay was used to detect the migration and invasion ability in MDA-MB-231 and MDA-MB-468 cells with *METTL3* transient overexpression and *METTL3* overexpression rescued with cycloleucine (left panels). Relative fold change was shown as the proportion of the number of control cells transfected with pcDNA3.1-FLAG (right panels). Original magnification, 100×. **(D)** Adhesion assay was used to detect the adhesion ability of MDA-MB-231 and MDA-MB-468 cells with *METTL3* transient overexpression (left panels). Relative fold change was shown as the proportion of the number of control cells transfected with pcDNA3.1-FLAG (right panels). Original magnification, 100×. *P < 0.05, **P < 0.01. Error bars represent the mean ± SD of three independent experiments.

For the knockdown of *METTL3*, three siRNAs targeted to *METTL3* were transfected according to the manufacturer's instructions (Figures 2A,B). Two sequences, si-*METTL3*-1604 (KD2) and si-*METTL3*-1126 (KD3), were selected for subsequent experiments. To investigate whether *METTL3* could inhibit TNBC metastasis, the effect of *METTL3* on migration, invasion, and adhesion to cell-extracellular matrix (ECM) was detected by trans-well assay or adhesion assay in TNBC cell lines, MDA-MB-231, and MDA-MB-468. The results showed that *METTL3* knockdown (KD) significantly increased the ability of migration and invasion, as well as the adhesion capability to ECM in both MDA-MB-231 and MDA-MB-468 cells (Figures 2C–E), indicating that *METTL3* could inhibit the potential of cell mobility of TNBC cells. Then, to determine whether *METTL3*-inhibited potential of cell mobility was related to m6A modification or not, *METTL3* was transiently overexpressed in MDA-MB-231 and MDA-MB-468 cells followed by the treatment with cycloleucine, a small molecule inhibitor of m6A modification. The results of trans-well assays demonstrated that *METTL3* overexpression (OE)-suppressed migration, invasion, and adhesion were significantly recovered by cycloleucine (Figures 3A–D). These results strongly suggested that *METTL3* inhibited the potential of cell mobility of TNBC cells by enhancing m6A modification.

### *COL3A1* Was Identified as a Potential Target of *METTL3* in TNBC

**Figure 4:**
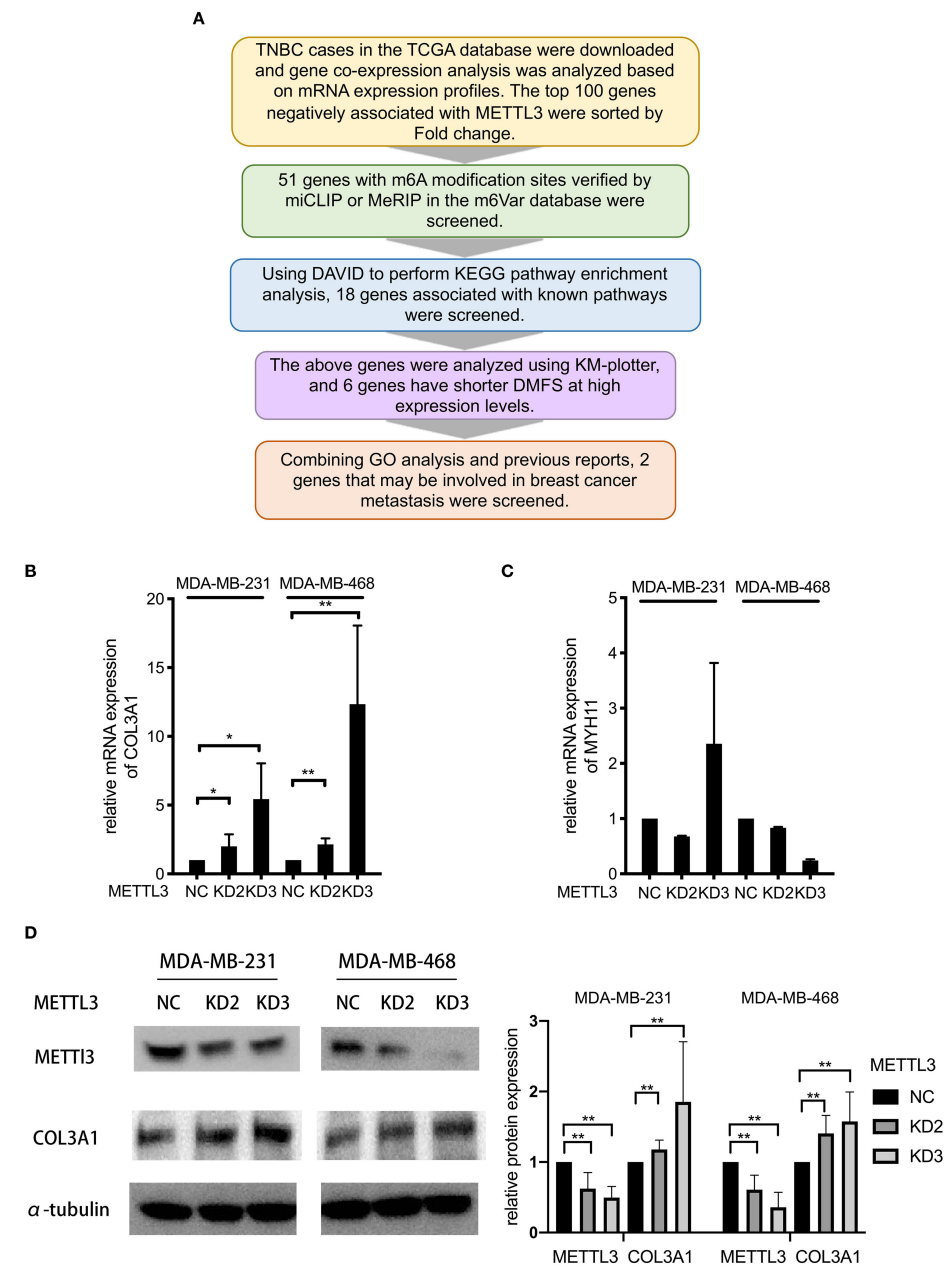
*COL3A1* was identified as a potential target of *METTL3* in TNBC. **(A)** Flowchart for screening potential target genes. **(B,C)** qRT-PCR was used to detect *COL3A1* and *MYH11* expression in MDA-MB-231 and MDA-MB-468 cells transfected with the si-NC or the si-*METTL3*. 18S was used as an internal control. **(D)** Western blot was used to detect *COL3A1* expression in MDA-MB-231 and MDA-MB-468 cells transfected with the si-NC or the si-*METTL3*. α-tubulin was used as a loading control (left panel). The blots were scanned and the abundance assessed quantitatively using ImageJ (right panel). *P < 0.05, **P < 0.01. Error bars represent the mean ± SD of three independent experiments.

It was known that m6A modification could down-regulate gene expression by accelerating RNA degradation (12–14). Therefore, to identify the target gene of *METTL3* involved in *METTL3*-inhibited metastasis, multi-step screening was performed as summarized in Figure 4A. Firstly, mRNA expression profiles of 91 TNBC patients in the TCGA dataset were downloaded, and the differentially expressed genes (DEG) were screened to identify those with *P* < 0.05 and log fold change (FC) using the “limma” package in R. The Log FC of DEG genes more than 0 was identified as representing positive related genes, whereas that <0 represented negative related genes. Among the genes for which mRNA expression was negatively related to *METTL3*, the top 100 genes according to the correlation coefficient were selected for further m6A methylation analysis using the m6Avar database (http://m6avar.renlab.org). All these genes and related information were listed in Table S1. As a result, 51 genes, which were verified to be able to be modified by m6A, were screened. Then, with the KEGG pathway enrichment analysis by DAVID (https://david.ncifcrf.gov), 18 genes were shown to be associated with the focal adhesion pathway, metabolism pathway, and so on, suggesting close involvement with metastasis in breast cancer. Subsequently, the association of 18 genes with the DMFS of TNBC patients was further analyzed using KM-plotter, and six alternative genes were found to have shorter DMFS at high levels in TNBC (Table 1). In particular, *COL3A1* and *MYH11* aroused our attention, because, according to the Gene Ontology (GO) analysis in DAVID, it was shown that *COL3A1* was involved in the biological process of skeletal system development and cell-matrix adhesion, and *MYH11* was involved in the biological process of elastic fiber assembly, which were similar to the findings of previous studies that they could promote metastasis in breast cancer. Therefore, the two genes were chosen as target gene candidates for *METTL3*. The further verification result of qRT-PCR detection showed that *METTL3*-KD only up-regulated the mRNA expression of *COL3A1*, but not *MYH11* in MDA-MB-231 and MDA-MB-468 cells (Figures 4B,C). Similarly, the result of western blot assay also confirmed that *METTL3*-KD increased the protein level of *COL3A1* in TNBC cells (Figure 4D). All of the above data strongly suggested that *COL3A1* might be the target gene of *METTL3*.

**Table 1:** 18 genes which were associated with pathways involved in metastasis and relative KEGG pathway, and DMFS analysis.

	**Gene name**	**KEGG Pathway enrichment analysis**	**DMFS analysis** **HR (*P*)**
1	*FZD4*	Wnt signaling pathway, Hippo signaling pathway, signaling pathways regulating pluripotency of stem cells, melanogenesis, HTLV-I infection, pathways in cancer, proteoglycans in cancer, basal cell carcinoma	0.47 (0.034)
2	*UTS2R*	Neuroactive ligand-receptor interaction	1.42 (0.350)
3	*PTPRJ*	Adherens junction	1.66 (0.048)
4	*CPSF3*	mRNA surveillance pathway	0.50 (0.088)
5	*MTHFD2L*	One carbon pool by folate, Metabolic pathways	1.77 (0.035)
6	*MYH11*	Tight junction	2.06 (0.033)
7	*LPGAT1*	Glycerophospholipid metabolism	1.57 (0.270)
8	*LAPTM4B*	Lysosome	1.36 (0.230)
9	*IL1R1*	MAPK signaling pathway, cytokine–cytokine receptor interaction, NF-kappa B signaling pathway, osteoclast differentiation, hematopoietic cell lineage, inflammatory mediator regulation of TRP channels, amoebiasis, HTLV-I infection	1.59 (0.160)
10	*COL3A1*	PI3K-Akt signaling pathway, focal adhesion, ECM-receptor interaction, platelet activation, protein digestion and absorption, amoebiasis	1.74 (0.041)
11	*ALG10B*	N-Glycan biosynthesis, metabolic pathways	2.14 (0.033)
12	*GMPPB*	Fructose and mannose metabolism, amino sugar and nucleotide sugar metabolism, metabolic pathways	1.43 (0.190)
13	*RNASEH1*	DNA replication	1.53 (0.100)
14	*UBE4B*	Ubiquitin mediated proteolysis, protein processing in endoplasmic reticulum	2.46 (0.007)
15	*NCOA1*	Thyroid hormone signaling pathway	1.47 (0.200)
16	*SNRPE*	Spliceosome	1.45 (0.160)
17	*TGM2*	Huntington's disease	0.54 (0.043)
18	*FDFT1*	Steroid biosynthesis, metabolic pathways, biosynthesis of antibiotics	1.28 (0.340)

### *METTL3* Down-Regulated the Expression of *COL3A1* by Increasing m6A Level

**Figure 5:**
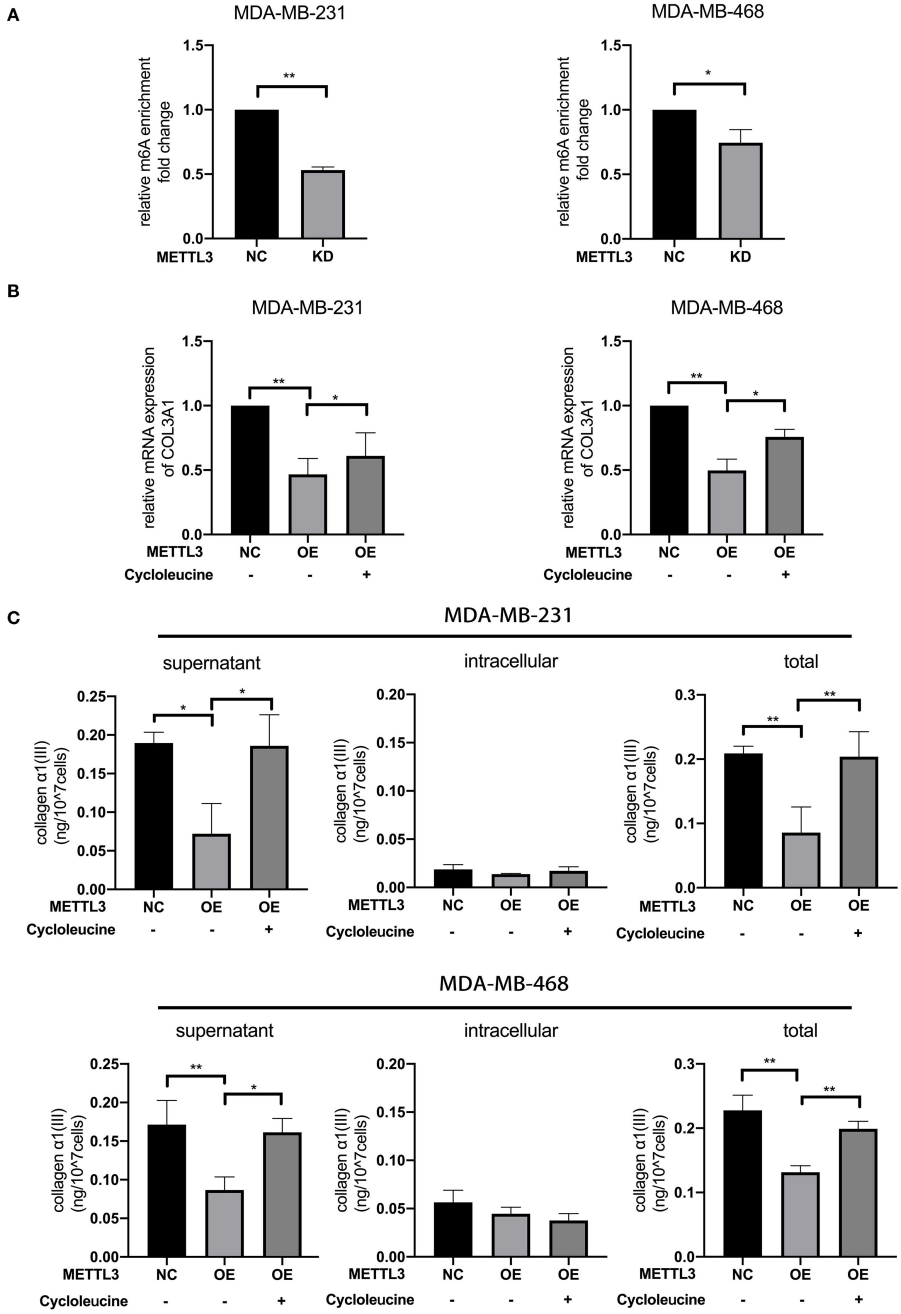
*METTL3* down-regulated the expression of *COL3A1* by increasing m6A levels. **(A)** MeRIP-qRT-PCR was used to detect the m6A modification level of *COL3A1* in MDA-MB-231 and MDA-MB-468 cells transfected with si-NC or si-*METTL3*. The relative enrichment fold changes were shown as proportions of control cells enrichment. **(B)** qRT-PCR was used to detect *COL3A1* expression in MDA-MB-231 and MDA-MB-468 cells with *METTL3* transient overexpression or the combination of *METTL3* overexpression with cycloleucine. 18S was used as an internal control. **(C)** ELISA was used to detect the secretion level of collagen α1 (III) of the supernatant (left panels), intracellular (middle panels) and total secreted protein (right panels) in MDA-MB-231 and MDA-MB-468 cells with *METTL3* transient overexpression or the combination of *METTL3* overexpression with cycloleucine. *P < 0.05, **P < 0.01. Error bars represent the mean ± SD of three independent experiments.

To investigate whether *COL3A1* was regulated by *METTL3*-mediated m6A methylation or not, the relative m6A enrichment level change of *COL3A1* before and after *METTL3*-KD was detected by MeRIP-qRT-PCR in MDA-MB-231 and MDA-MB-468 cells. As shown in Figure 5A, *METTL3*-KD significantly reduced m6A-methylated *COL3A1* mRNA expression. Furthermore, with qRT-PCR detection, it was shown that *METTL3*- OE decreased the mRNA expression of *COL3A1*, while cycloleucine partially recovered *METTL3*-OE-down-regulated *COL3A1* (Figure 5B). As shown in Figure 5C, the results of ELISA showed that the secretion level of Collagen α1(III) in supernatant decreased by *METTL3*-OE could be recovered by cycloleucine while the *METTL3*-OE decreased the intracellular level of Collagen α1(III), the cycloleucine could not recover the reduction of the intracellular level of Collagen α1(III). Considering the amount of Collagen α1(III) secreted into the supernatant is greater than the amount in the cell, the total levels of Collagen α1(III) which were decreased by *METTL3*-OE could be recovered by cycloleucine after normalizing (Figure 5C). These results proved that *COL3A1* was down-regulated by *METTL3*-mediated m6A modification on *COL3A1* (Figure 5C).

### *COL3A1* Promoted the Metastatic Ability of TNBC Cells

**Figure 6:**
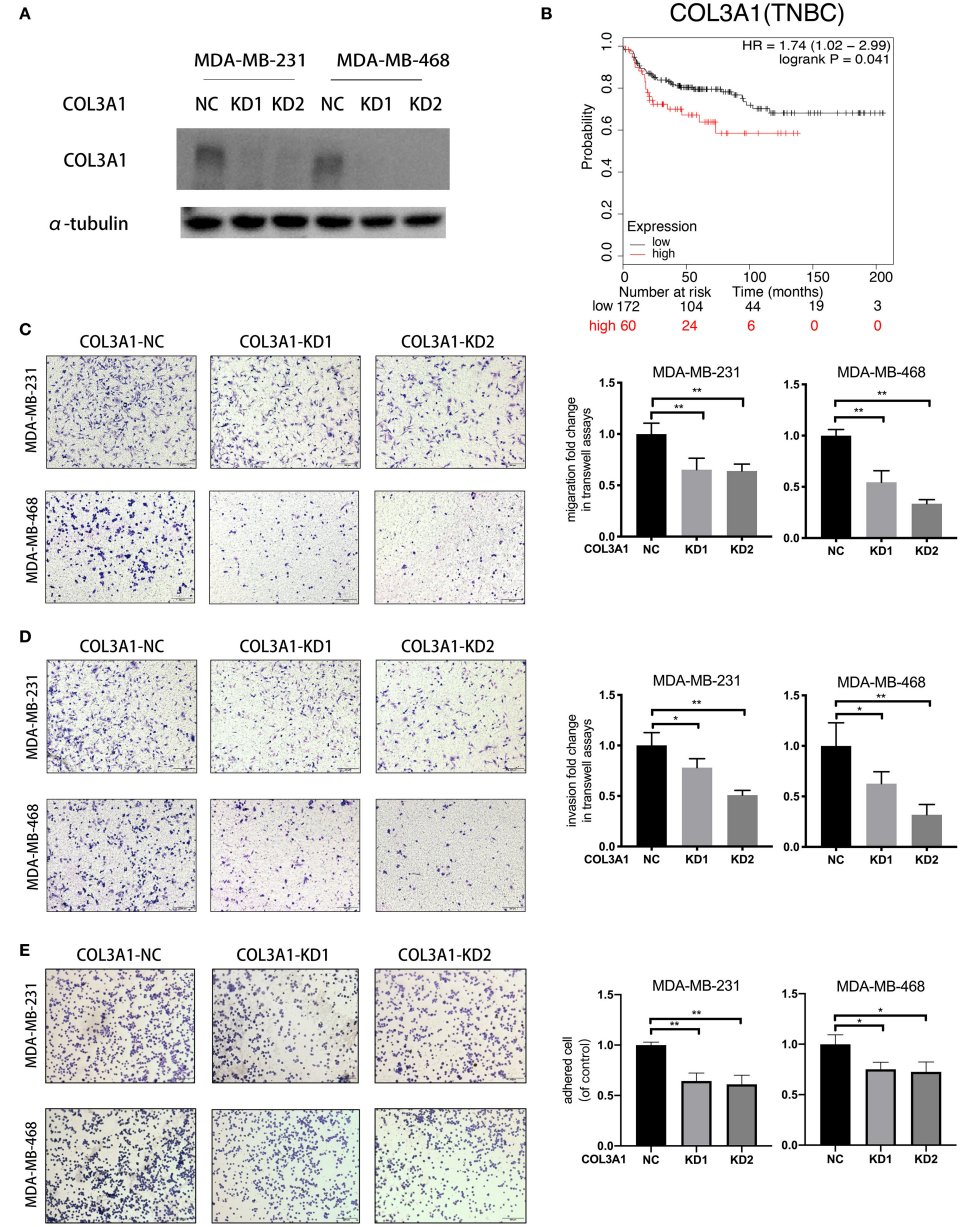
*COL3A1* promoted the metastatic ability of TNBC cells. **(A)** Western blot was used to detect *COL3A1* expression in MDA-MB-231 and MDA-MB-468 cells transfected with the si-NC or the si-*COL3A1*. α-tubulin was used as a loading control. **(B)** Kaplan–Meier analysis for the DMFS of *COL3A1* in TNBC patients using KM-plotter online database. **(C,D)** The migration and invasion of MDA-MB-231 and MDA-MB-468 with transient *COL3A1*-KD was detected by transwell assays (left panels). Relative fold change was shown as the proportion of the number of control cells transfected with si-NC (right panels). Original magnification, 100×. **(E)** The adhesion ability of MDA-MB-231 and MDA-MB-468 after transient *COL3A1*-KD was evaluated by adhesion assay (left panels). Relative fold change was shown as the proportion of the number of control cells transfected with si-NC (right panels). Original magnification, 100×. *P < 0.05, **P < 0.01. Error bars represent the mean ± SD of three independent experiments.

The role of *COL3A1* in TNBC metastasis was further investigated. The influence of *COL3A1* on DMFS of TNBC patients analyzed by KM-plotter is shown in Figure 6B, the DMFS of TNBC patients with high-expression *COL3A1* was shorter than in those with a low expression thereof. There was no significant difference in the expression level of *COL3A1* and DMFS in the overall patients (Figure S1). In addition, when *COL3A1* was knocked-down in MDA-MB-231 and MDA-MB-468 cells (Figure 6A), the migration, invasion, and adhesion to ECM were significantly suppressed (Figures 6C–E). These data indicated that *COL3A1* played an important role in promoting metastasis in TNBC.

### Reduced *COL3A1* m6A Modification by *METTL3* Inhibition Leads to Poor Prognosis in TNBC Patients

**Figure 7:**
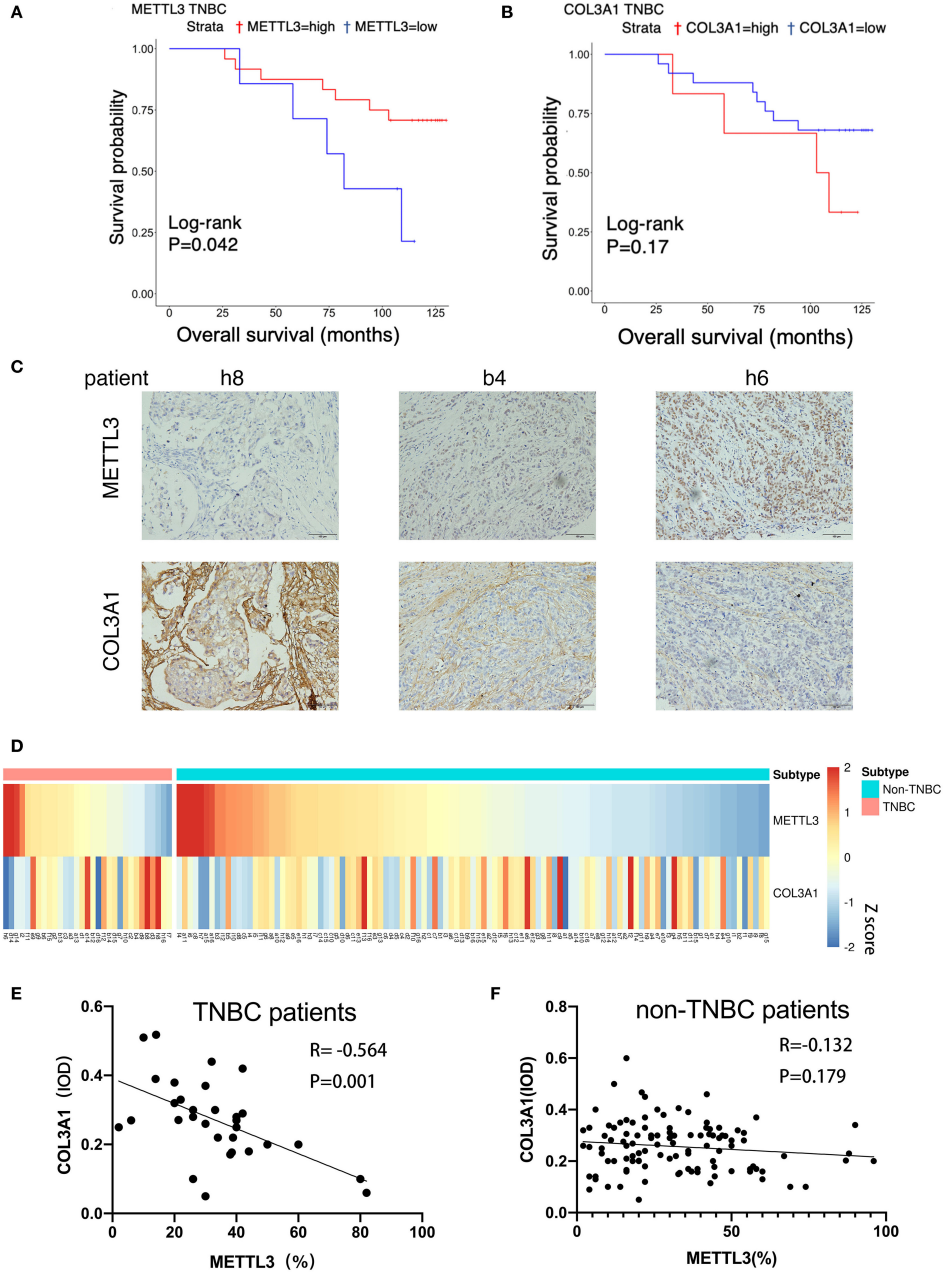
The correlation between *METTL3* and *COL3A1* in breast cancer patients. **(A,B)** Kaplan–Meier analysis for the OS of *METTL3* and *COL3A1* in TNBC patients of the TMA sections. **(C)** The expression of *METTL3* and *COL3A1* detected by IHC in the representative samples of breast cancer. h8, low expression of *METTL3* and high expression of *COL3A1*. b4, middle expression of *METTL3* and *COL3A1*. h6, high expression of *METTL3* and low expression of *COL3A1*. Original magnification, 200×. **(D)** Heatmap of the expression level of *METTL3* and *COL3A1* protein in breast cancer patients. **(E,F)** Correlation of *METTL3* and *COL3A1* in human TNBC patients (*n* = 31) and non-TNBC patients (*n* = 109), respectively, in TMA sections.

In order to verify the effects of *METTL3* and *COL3A1* on the prognosis of breast cancer patients *in vivo*, the expression of *METTL3* and *COL3A1* was investigated by immunohistochemistry using TMA sections containing 31 TNBC patients and 109 Non-TNBC patients. The results of survival analysis showed that low *METTL3* expression was related to short overall survival (OS) (Figure 7A) in TNBC but not in non-TNBC (Figure S2A). The OS of TNBC with *COL3A1*-high expression was shorter than that with low expression (Figure 7B), which may not be statistically significant because of the small number of TNBC cases. In non-TNBC, *COL3A1* has the opposite trend, and patients with high expression have longer OS (Figure S2B). Finally, the relationship between *METTL3* and *COL3A1* in breast cancer was analyzed with Pearson correlation analysis (Figures 7C,D). The results showed that the expression level of *COL3A1* was negatively correlated with *METTL3* expression in TNBC patients (*R* = −0.564, *P* = 0.001; Figure 7E); however, the expression level of *METTL3* and *COL3A1* had no significant relationship in the 109 NTNBC patients (*R* = −0.132, *P* = 0.170; Figure 7F). This result further confirmed that the metastasis-inhibition function of *METTL3* by negatively regulating *COL3A1* expression was TNBC specific.

## Discussion

In this study, by analyzing the prognostic role of m6A modulators (*METTL3, METTL14, WTAP, FTO*, and *ALKBH5*) in breast cancer using on-line databases, we found that only *METTL3* played an important role in TNBC metastasis. The expression of *METTL3* in breast cancer tissues was lower than that in normal tissues, and *METTL3* was a protective factor of DMFS in TNBC. Using TNBC cell lines, it was confirmed that *METTL3* could inhibit metastasis by increasing the level of m6A modification, and *COL3A1* was identified as one of the possible target genes of *METTL3*. Furthermore, reduced expression of *METTL3* was proved to be able to contributes the potential of mobility of triple-negative breast cancer cells by m6A methylation-mediated *COL3A1* up-regulation.

The role of m6A modification that is mainly regulated by methyltransferases and demethylases, is complicated and specific in various cancers. A bioinformatics analysis study involving 33 cancers showed that m6A modulators are closely related to both the activation pathway and inhibition pathway of cancer; the distribution of m6A modifications varies widely among different cancers; even for the same type of cancer, the prognostic function of m6A was not consistent within each sub-type (15). Several studies also exhibit the complicated roles of m6A modulators in the development of breast cancer. It was reported that *FTO*, a key m6A demethylase, was up-regulated and significantly associated with poor prognosis in breast cancer (8); *FTO*-reduced m6A modification could promote breast cancer cell proliferation and metastasis by inhibiting BNIP3 expression (11). Similarly, *METTL14* overexpression or *ALKBH5* silence could also inhibit the growth and migration of breast cancer cell line, MDA-MB-231 (16). The opposite result was also reported such that the deficiency of *METTL3* could inhibit the proliferation of breast cancer cell line MCF-7, by m6A-level-decreasing-mediated Bcl-2 up-regulation (10). In this study, using online database, we analyzed the prognostic role of five m6A modulators in breast cancer, especially in TNBC, the sub-type with the worst prognosis and the greatest potential for metastasis, and found that *METTL3* is the most critical in TNBC, that *METTL3* occurred at low expression in TNBC, and was a protective factor of DMSF. The results of the TMA section also confirm the protective effect of *METTL3* on the overall survival of TNBC patients. These results are consistent with some previous researches (16), while contradictory to the other researches focused on proliferation (10, 17). This difference might be due to the different subtypes of the breast cancer cell lines used. It should be taken into consideration that those previous study had mainly used non-TNBC cell lines. Meanwhile, the conclusion of previous studies was based on the result of cellular level investigation and lack of clinical specimen validation. Therefore, although the trend in m6A modification levels was consistent, different regulators might eventually cause opposite effects by regulating different target genes; an m6A modulator might also execute different functions in each sub-type due to the heterogeneity of cancer. The function of *METTL3* and other m6A modulators in other sub-types of breast cancer warrants further investigation in the future.

Widely distributed in eukaryotes, RNA methylation modification occurs in thousands of genes (18). *METTL3* is known to achieve its biological effects by increasing the m6A modification level of target genes, which leads to various effects on target genes, such as faster degradation of target gene mRNA, increase in target gene translation efficiency, or accurate cell localization of target genes (2–5). Among them, the mechanism of accelerating the rate of degradation of target gene mRNA is most widely investigated. Therefore, *METTL3* might have many target genes in TNBC, and the inhibitory effect of *METTL3* on TNBC metastasis might also be achieved by affecting multiple target genes together. In this study, by expression correlation analysis and methylation search, *COL3A1* was identified as the target gene candidate of *METTL3*. Collagen type III alpha 1 chain (*COL3A1*), which encodes the pro-alpha 1 chains of type III collagen, previously was reported to be associated with malignant potential of breast cancer (19). To date, no specific mechanism has been reported for *COL3A1* regulation. In this study, we proved that knocking down *METTL3*, while reducing the methylation of m6A, also eventually up-regulated the expression level of *COL3A1*. Validation of clinical specimens indicated that this relationship appears to be only in TNBC patients. Thus, this study demonstrated that *METTL3* and *COL3A1* might only play a significant role in the TNBC subtype. Certainly, there must be multiple target genes of *METTL3* that play the same role. *COL3A1* may also have modification sites different from those provided by online databases. Further MeRIP-sequence is needed to clarify the mechanisms of *METTL3* in metastasis inhibition of TNBC in the future and the specific modified sites of *COL3A1*. Considering that the mRNA level of *COL3A1* has changed, we speculate that the change of m6A level may affect the degradation rate of *COL3A1* mRNA in TNBC cells. In this case, the position of m6A seems to be more likely to be distributed in the 3′UTR region of mRNA (2). But this speculation still needs further experimental proof.

Collagen, the most abundant component of extracellular matrix (ECM) in the tumor micro-environment, is known to be able to contribute to tumor progression (20). Collagen could promote the metastasis and proliferation of cancer by increasing the accumulation of integrin, which leads to phosphorylation of focal adhesion kinase and activation of extracellular signal-regulated kinase (21). *COL3A1*, which encodes pro-alpha1 chains of type III collagen, could form homotrimeric fibrils to play its role. Except for normal localization in connective tissues, *COL3A1* was also found to be highly expressed in various cancers including bladder cancer, glioblastoma, and gastric cancer (22–24). In breast cancer, it was reported that stromal *COL3A1* expression was significantly increased from benign breast tumors to malignant breast tumors (18). Another study has shown that when Pirfenidone, an anti-fibrotic drug, was applied to breast cancer to investigate its possible role on tumor microenvironment normalization, the level of *COL3A1* was down-regulated, thereby inhibiting the *TGF*β signaling pathway. That causes the reduction of extracellular matrix components, which significantly increases vascular function and perfusion, and increases the anti-tumor efficacy of doxorubicin (25). Therefore, these studies showed that *COL3A1* played an important role in the development of breast cancer. In addition, it was reported that *COL3A1* up-regulation cause extracellular matrix changes and reduced tumor perfusion, while the hypoxic micro-environment caused by hypoperfusion was considered to be the main reason for forcing cancer cells to metastasize (26). Therefore, the reduction of tumor perfusion caused by up-regulation of *COL3A1* was likely to be one of the reasons for the increased ability of TNBC cells to metastasis. In this study, we demonstrated that *COL3A1*, which was up-regulated by the reduced expression of *METTL3*, could contribute to TNBC metastasis. The molecular mechanism of *COL3A1* in promoting TNBC metastasis warrants further investigation.

In summary, this study not only revealed that, among m6A modulators, only *METTL3* played an important role in TNBC metastasis, but also demonstrated that the low expression of *METTL3*-reduced m6A modification could promote TNBC metastasis by up-regulating its target gene, *COL3A1*. Our results provided sufficient evidence of the important epigenetic role in the development of TNBC and allowed a more comprehensive understanding of the mechanism of tumor metastasis. *METTL3* and its target gene *COL3A1* might have the potential to become novel biomarkers for TNBC prognostic prediction and new targets for TNBC therapy.

## Data Availability Statement

Publicly available datasets were analyzed in this study. This data can be found here: https://www.cancer.gov/?TCGA-BRCA&lt;/b&gt.

## Ethics Statement

This study was approved by the Ethics Committee of Shanghai Outdo Biotechnology Company (YB M-05-02), and all patients have signed informed consent. The patients/participants provided their written informed consent to participate in this study.

## Author Contributions

YS and CZ analyzed and interpreted the data regarding the m6A modulators and DMFS of breast cancer patients, performed most experiments and were major contributor in writing the manuscript and contributed equally to this work. YJ and DW performed partial qRT-PCR experiment. BB analyzed the correlation analysis between *METTL3* and other genes. JF helped evaluated the expression of *METTL3* in IHC. ST helped with the making of heatmap. XQ and YL provided guidance on interpreting the results. XC and YT designed the study and revised the manuscript. All authors read and approved the final manuscript.

## Conflict of Interest

The authors declare that the research was conducted in the absence of any commercial or financial relationships that could be construed as a potential conflict of interest.

## Abbreviations

*ALKBH5*: AlkB family member 5
*COL3A1*: collagen type III alpha 1 chain
DMFS: the distance-metastasis-free survival
ECM: cell-extracellular matrix
FC: fold change
GO: Gene Ontology
GTEx: the Genotype Tissue Expression
IHC: Immunohistochemistry
KD: knockdown
*METTL3*: methyltransferase-like 3
*METTL14*: methyltransferase-like 14
*MYH11*: myosin heavy chain 11
NC: negative control
OE: overexpression
PVDF: polyvinylidene difluoride
qRT-PCR: quantitative real-time
TCGA: the Cancer Genome Atlas
TNBC: triple-negative breast cancer
TMA: The tissue microarray
*WTAP*: Wilms tumor 1-associated protein.
