# Supplementary figures and images for: Reduced Expression of METTL3 Promotes Metastasis of Triple-Negative Breast Cancer by m6A Methylation-Mediated COL3A1 Up-Regulation
Source: Front Oncol. 2020 Jul 14;10:1126. doi: 10.3389/fonc.2020.01126 (PMC7381173; doi:10.3389/fonc.2020.01126)

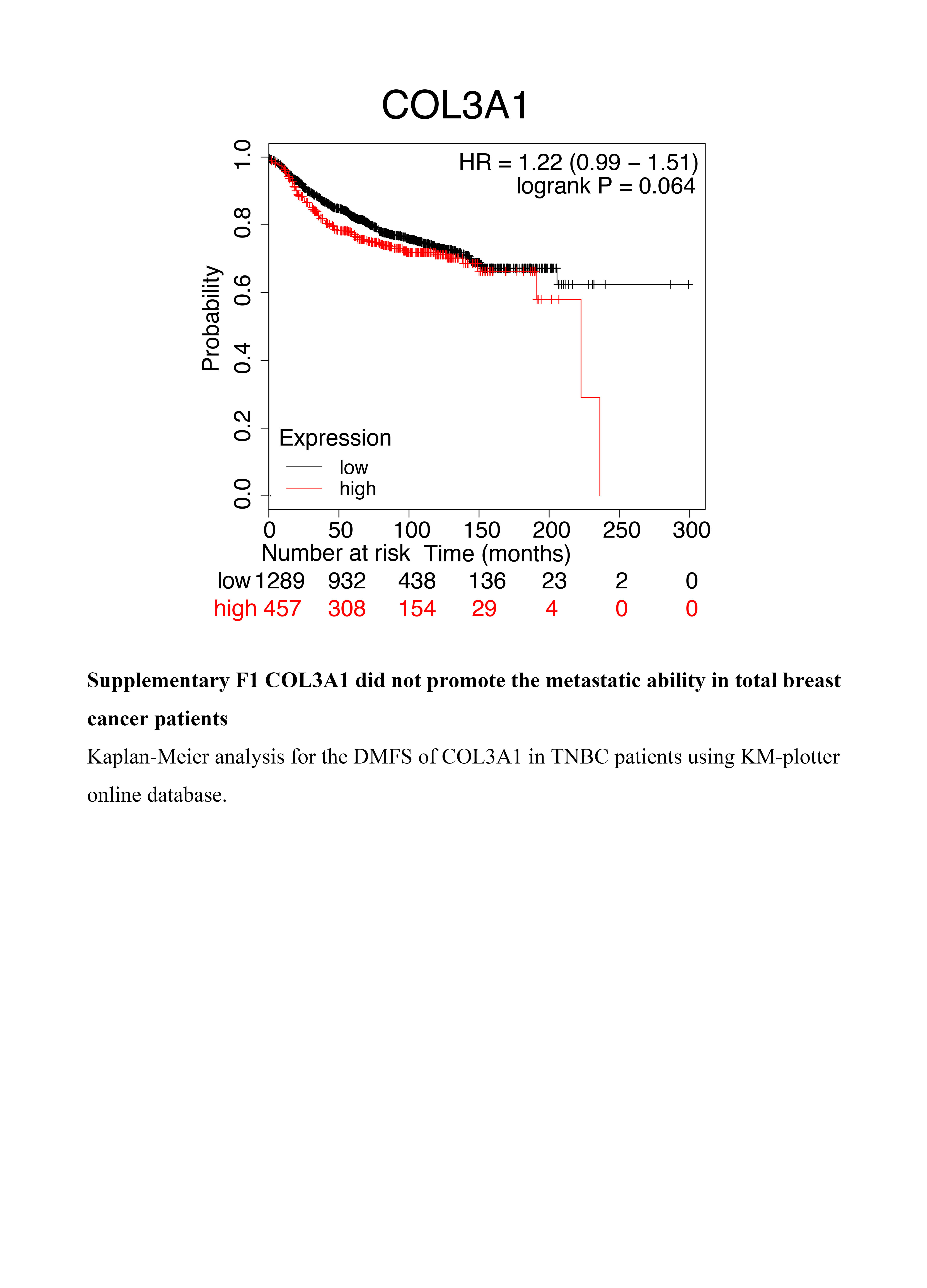

Supplement: Supplementary file 2 [file Image_1.JPEG]

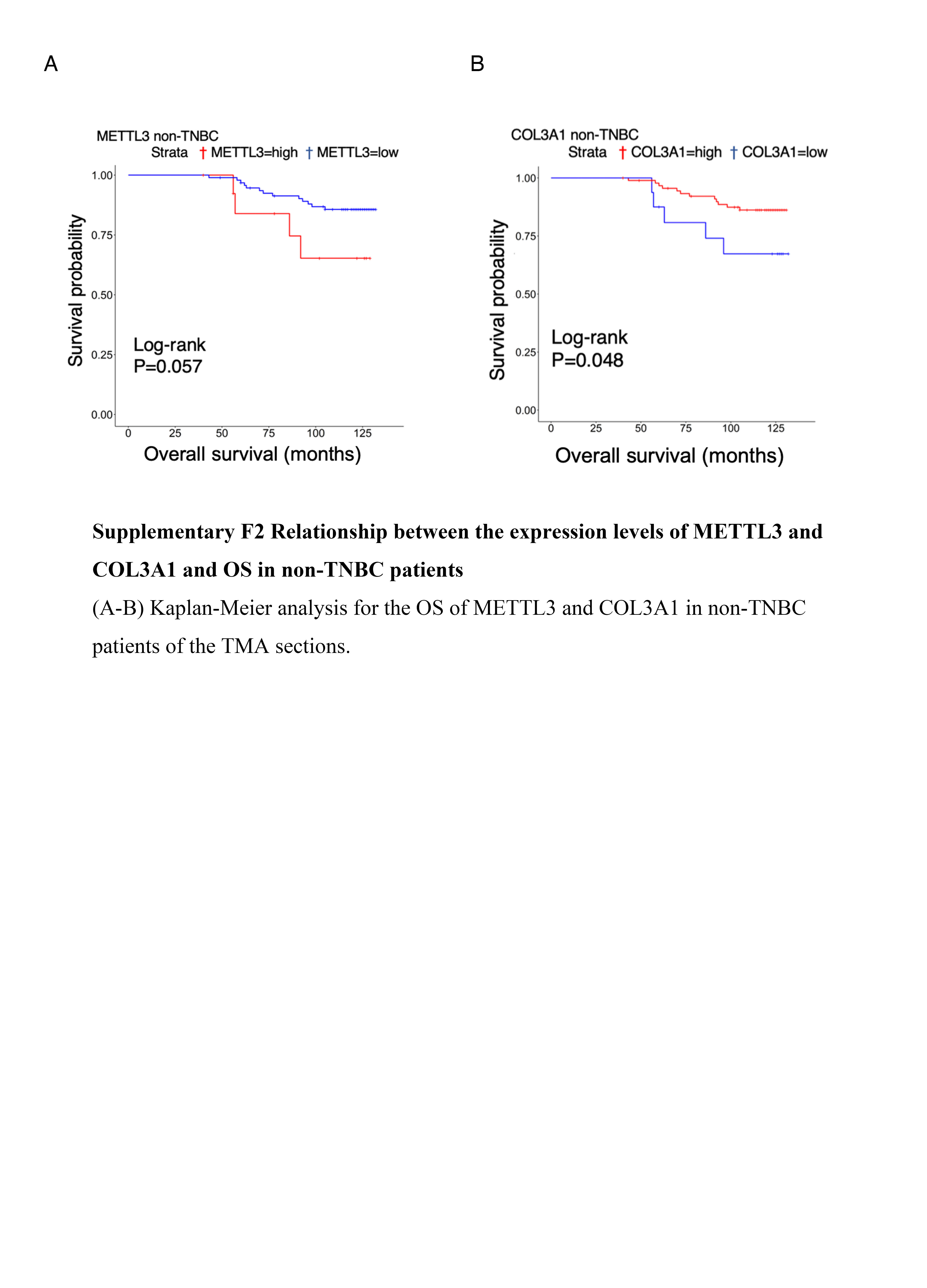

Supplement: Supplementary file 3 [file Image_2.JPEG]
